# Production of Interferons and β-Chemokines by Placental Trophoblasts of HIV-1-Infected Women

**DOI:** 10.1155/S1064744901000175

**Published:** 2001

**Authors:** Bang-Ning Lee, Hunter Hammill, Edwina J. Popek, Stanley Cron, Claudia Kozinetz, Mary Paul, William T. Shearer, James M. Reuben

**Affiliations:** 1 Department of Molecular Pathology Division of Pathology and Laboratory Medicine Box 7 The University of Texas M.D. Anderson Cancer Center, 1515 Holcombe Blvd. Houston TX 77030 USA; 2 The Women's Hospital of Texas Houston TX USA; 3 Department of Pathology Texas Children's Hospital Houston TX USA; 4 Department of Pediatrics Texas Children's Hospital Houston TX USA

## Abstract

*Objective:* The mechanism whereby the placental cells of a human immunodeficiency virus (HIV)-1-infected mother protect the fetus from HIV-1 infection is unclear. Interferons (IFNs) inhibit the replication of viruses by acting at various stages of the life cycle and may play a role in protecting against vertical transmission of HIV-1. In addition the β-chemokines RANTES (regulated on activation T cell expressed and secreted), macrophage inflammatory protein-1-α (MIP-1α), and MIP-1β can block HIV-1 entry into cells by preventing the binding of the macrophage-trophic HIV-1 strains to the coreceptorCCR5. In this study the production of IFNs and β-chemokines by placental trophoblasts of HIV-1-infected women who were HIV-1 non-transmitters was examined.

*Methods:* Placental trophoblastic cells were isolated from 29 HIV-1-infected and 10 control subjects. Supernatants of trophoblast cultures were tested for the production of IFNs and β-chemokines by enzyme linked immunosorbent assay (ELISA). Additionally, HIV-1-gag and IFN-β transcripts were determined by a semi-quantitative reverse transcription polymerase chain reaction (RT-PCR) assay.

*Results:* All placental trophoblasts of HIV-1-infected women contained HIV-1-gag transcripts. There were no statistical differences in the median constitutive levels of IFN-α and IFN-γ produced by trophoblasts of HIV-1- infected and control subjects. In contrast, trophoblasts of HIV-1-infected women constitutively produced significantly higher levels of IFN-β protein than trophoblasts of control subjects. Furthermore, the median levels of β-chemokines produced by trophoblasts of HIV-infected and control women were similar.

*Conclusions:* Since there was no correlation between the placental HIV load and the production of interferons or β-chemokines, the role of trophoblast-derived IFNs and β-chemokines in protecting the fetus from infection with HIV-1 is not clear.


### Production of interferons and β-chemokines by placental trophoblasts of HIV-1-infected women

**Bang-Ning Lee[1], Hunter Hammill[2], Edwina J. Popek[3], Stanley Cron[4], Claudia Kozinetz[4], Mary Paul[4], William T. Shearer[4] and James M. Reuben[1]**

*[1]Department of Molecular Pathology, The University of Texas M.D. Anderson Cancer Center, Houston, TX*
*[2]The Women's Hospital of Texas, Houston, TX*
*[3]Department of Pathology and [4]Department of Pediatrics, Texas Children's Hospital, Houston, TX*

***Objective:*** The mechanism whereby the placental cells of a human immunodeficiency virus (HIV)-1-infected mother protect the fetus from HIV-1 infection is unclear. Interferons (IFNs) inhibit the replication of viruses by acting at various stages of the life cycle and may play a role in protecting against vertical transmission of HIV-1. In addition the β-chemokines RANTES (regulated on activation T cell expressed and secreted), macrophage inflammatory protein-1-α (MIP-1α), and MIP-1β can block HIV-1 entry into cells by preventing the binding of the macrophage-trophic HIV-1 strains to the coreceptor CCR5. In this study the production of IFNs and β-chemokines by placental trophoblasts of HIV-1-infected women who were HIV-1 non-transmitters was examined.

***Methods:*** Placental trophoblastic cells were isolated from 29 HIV-1-infected and 10 control subjects. Supernatants of trophoblast cultures were tested for the production of IFNs and β-chemokines by enzyme linked immunosorbent assay (ELISA). Additionally, HIV-1-*gag* and IFN-β transcripts were determined by a semi-quantitative reverse transcription polymerase chain reaction (RT-PCR) assay.

***Results:*** All placental trophoblasts of HIV-1-infected women contained HIV-1-*gag* transcripts. There were no statistical differences in the median constitutive levels of IFN-α and IFN-γ produced by trophoblasts of HIV-1-infected and control subjects. In contrast, trophoblasts of HIV-1-infected women constitutively produced significantly higher levels of IFN-β protein than trophoblasts of control subjects. Furthermore, the median levels of β-chemokines produced by trophoblasts of HIV-infected and control women were similar.

***Conclusions:*** Since there was no correlation between the placental HIV load and the production of interferons or β-chemokines, the role of trophoblast-derived IFNs and β-chemokines in protecting the fetus from infection with HIV-1 is not clear.

Key words: NON-TRANSMITTERS, IMMUNE REGULATORS, RANTES, MIP-1α, MIP-1β

The mechanism by which HIV-1 infection is transmitted from a mother to her infant is unclear but may be related to a number of risk factors, including preterm labor, the interval between membrane rupture and delivery, and whether the mother breastfeeds or is infected with a sexually-transmitted disease during pregnancy[1]. At the cellular level vertical transmission of HIV-1 may occur as a result of an infected maternal cell crossing from the maternal circulation into the fetal circulation or by the direct transfer of the virus from a maternal cell to a fetal cell[2]. For example, it has been suggested that maternal macrophage-trophic virus may serve as a vehicle to infect the

Correspondence to: James M. Reuben, PhD, Department of Molecular Pathology, Division of Pathology and Laboratory Medicine, Box 7, The University of Texas M.D. Anderson Cancer Center, 1515 Holcombe Blvd., Houston, TX 77030.
E-mail: jreuben@mdanderson.org fetus via infection of placental macrophages[3,4]. Placental macrophages become infected with HIV-1 through the CD4 receptor[5,6] and the CCR5 coreceptor[7]. However, the susceptibility of placental trophoblasts to infection with HIV-1 is controversial: both successful[2,4,8] and unsuccessful[9,10] infections have been reported. In support of the former, we[11] and others[2,4,12,13] have found HIV-1 provirus in macrophages and trophoblasts and HIV-1-*gag* transcripts in the placental trophoblasts from women infected with HIV-1[14]. Nonetheless, the replication rate of HIV-1 in trophoblasts is low[15,16] and may not permit dissemination of the infection to other placental cells.

Immunological factors increasing the risk of maternal-to-fetal HIV-1 transmission include maternal immunosuppression[17] and inflammation of the placental membranes, mainly mediated by cytokines[18,19]. Placental macrophages and trophoblasts constitutively secrete a variety of cytokines, including granulocyte-macrophage colony stimulating factor, colony-stimulating factor, interleukin (IL)-1$\beta$, IL-6, tumor necrosis factor-$\alpha$ (TNF-$\alpha$), interferons, and transforming growth factor[20]. That some cytokines can up-regulate HIV-1 expression in T cells[21] and in a promonocytic cell line[22] has been demonstrated and may provide a mechanism through which fetal and maternal cells latently infected with HIV-1 become activated and release productive virus. Thus, it is tempting to speculate that the presence of inflammatory cytokines in the placental microenvironment plays a regulatory role in the expression of HIV-1. Even so, the presence of HIV-1-infected cells in the placenta and abnormal maternal immunologic characteristics do not always lead to vertical transmission[14]. While some workers believe that the suboptimal permissiveness of trophoblasts for HIV-1 replication may involve a restriction at the level of HIV-1 integration[13], others have speculated that factors influencing viral gene transcription and translation may be responsible[23,24]. Throughout pregnancy interferons (IFNs) can be detected in the maternal and fetal blood circulations in response to intrauterine infection with herpes simplex virus[25], albeit sporadically and at low concentration[26].

Monocyte-macrophage tropic HIV-1 isolates are non-syncytium inducing and are the predominant isolates transmitted from mother to fetus[27–29]. Placental macrophages express the CD4 receptor and the CCR3 and CCR5 chemokine coreceptors[7] that facilitate the entry of HIV-1 into the cell. Placental trophoblasts can be infected with HIV-1 even though they may express chemokine receptors[30], and not the CD4 receptor[8,12,30–32]. Chemokines on the other hand may prevent infection of maternal and fetal cells in the placenta by preventing the binding of HIV-1 to the chemokine receptors[33,34] on the surface of the susceptible cells. In this study the hypothesis that placental HIV-1 load, as measured by the level of HIV-1-*gag* transcripts, is inversely correlated with the levels of IFNs and chemokines produced by the trophoblasts was tested.

### SUBJECTS AND METHODS

### Study population

Thirty-nine women, of whom 29 were HIV-1-infected and ten were uninfected and healthy, agreed to particpate in this study, which was approved by the Institutional Research Board of the hospitals affiliated with the Harris County Hospital District in Houston, Texas. According to the census of the Texas Department of Health, the racial and ethnic distribution of parturient women in Harris County at the time of this study was 86% African-American, 5% Hispanic, and 9% other races. Among the 29 HIV-1-infected gravidas from whom term placentas were obtained at delivery, six were symptomatic for HIV-1 disease throughout pregnancy. Five of the symptomatic and 18 of the asymptomatic HIV-1-infected women received zidovudine (ZDV), either intrapartum or throughout pregnancy, or both (Table 1). CD4 T-cell counts were available for 22 of the HIV-1-infected women. Blood samples from 25 infants, including a set of twins, born to HIV-1-infected women were obtained at birth, 1 month, 6 months, and 12 months of age. The blood was assayed for the presence of HIV-1 infection by the co-culture method and/or for HIV-1 proviral DNA by the polymerase chain reaction (PCR). Enumeration of peripheral CD4+ T-cell counts of these infants was performed at birth. Five infants were lost to follow-up.

**Table 1** The level of HIV-1-*gag* transcripts in placental trophoblastic cells associated with the clinical characteristics of human immunodeficiency virus (HIV)-1-infected mothers at delivery, and the infants' status

| Sample number | Placental HIV-1 level* | *Mother's status* | | | *Infant's status* | |
|---|---|---|---|---|---|---|
| | | *ZDV*** | *Symptom*† | *CD4 count* | *HIV-1*†† | *CD4 count* |
| 1 | H | y1 | N | 195 | Neg | 2861 |
| 2 | H | no | Y | na | Neg | 722 |
| 3 | H | y1 | N | 537 | Neg | 4381 |
| 4 | H | no | N | na | LTFU | na |
| 5 | L | y1,2 | Y | na | Neg | 1987 |
| 6 | H | no | N | 814 | Neg | 4661 |
| 7 | H | y | N | 411 | Neg | 1728 |
| 8 | L | no | N | 284 | Neg | 1617/1802‡ |
| 9 | H | y | N | 499 | Neg | 3274 |
| 10 | L | no | N | 201 | LTFU | na |
| 11 | L | y1,2 | N | 390 | Neg | 2683 |
| 12 | L | y1,2 | N | 126 | Neg | 2969 |
| 13 | L | y1,2 | N | 364 | Neg | 3126 |
| 14 | L | y1,2 | Y | na | Neg | 2412 |
| 15 | L | no | N | 160 | LTFU | na |
| 16 | H | y | N | 336 | Neg | 5316 |
| 17 | H | y | N | na | Neg | 2576 |
| 18 | H | y | N | 330 | Neg | 3337 |
| 19 | L | y1,2 | Y | 403 | Neg | 2856 |
| 20 | H | y | N | 362 | Neg | 2941 |
| 21 | H | y | Y | 174 | Neg | 2028 |
| 22 | L | y | Y | na | Neg | 3444 |
| 23 | H | y | N | 327 | Neg | 4491 |
| 24 | H | y | N | 819 | LTFU | na |
| 25 | H | y | N | 212 | Neg | 850 |
| 26 | L | y | N | 123 | Neg | 2351 |
| 27 | L | y | N | na | LTFU | na |
| 28 | H | y | N | 10 | Neg | 3149 |
| 29 | H | y | N | 50 | Neg | 1736 |

*Levels of HIV-1-*gag* transcripts as determined by reverse transcription polymerase chain reaction (RT-PCR) (see Subjects and Methods): L, low (< 156 copies/μg of total RNA), H, high (> 156 copies/μg total RNA); **zidovudine (ZDV) administered: y = yes, but details not available; 1, ZDV administered throughout pregnancy; 2, ZDV administered intrapartum; †Y = symptomatic, N = asymptomatic; na, information not available; ††infant's status: Neg, HIV-1 negative, LTFU, lost to follow-up;‡a set of twins

Control placentas were collected from normal term deliveries performed by the same obstetrician who delivered the HIV patients. They were selected based on term gestation, with no medical complications of pregnancy or delivery. Delivery was vaginal or by scheduled repeat Cesarean section. The maternal ages of the control subjects and the HIV patients were similar.

### Isolation of placental trophoblastic cells

Trophoblastic cells were isolated from human term placentas of 36–40 weeks' gestation. Immediately after delivery rigorous isolation procedures[14,35–37] were employed to ensure the purity of the trophoblast preparations. Cell morphology and the production of human chorionic gonadotropin were determined in trophoblastic cell preparations using immunocytochemical staining as previously reported[14]. Cytospin preparations of the final trophoblastic cell fractions were stained with Wright–Giemsa medium. The trophoblast preparations consisted of a mixture of syncytiotrophoblasts and cytotrophoblasts, with < 3% leukocyte contamination.

### Production of interferons and β-chemokines by trophoblasts

Two ml aliquots of trophoblasts at a concentration of 2 x $10^6$ cells/ml in RPMI-1640 medium supplemented with L-glutamine, 10% human AB serum and antibiotics (200 U/ml penicillin and 200 μg/ml streptomycin) were placed in 12 mm × 75 mm tubes (Sigma Chemical Co., St. Louis, MO) and incubated at 37°C in a humidified atmosphere with 5% $CO_2$ for up to 48 h. At 12 h and 24 h the culture tubes were centrifuged at 450*g* for 10 min; the culture supernatants were harvested and stored frozen at -20°C until assayed for IFNs and β-chemokines. The levels of IFN-α, IFN-γ, macrophage inflammatory protein-1-α (MIP-1α), MIP-1β, and RANTES (regulated on activation T cell expressed and secreted) were determined in supernatants of trophoblastic cell cultures using Quantikine™ ELISA kits (R & D Systems, Minneapolis, MN). The level of IFN-β in supernatants of trophoblastic cell cultures was determined using the HuIFN-β1 ELISA kit (Toray-Fuji Bionics Inc., Tokyo, Japan). All measurements reported are the means of duplicate sampling.

### Reverse transcription of RNA into cDNA

Single-stranded cDNA in a final volume of 20 μl was prepared by reverse transcription using (RT) the GeneAmp RT-PCR kit (Applied Biosystems, Foster City, CA). Briefly, after denaturation of 1 μg freshly prepared RNA at 68°C for 10 min, a reaction mixture containing 2.5 μM random hexamers, 1 mM dNTP, 5 mM $MgCl_2$, 20 U RNase inhibitor, and 50 U murine leukemia virus (MuLV) reverse transcriptase was added to the sample tube. The reaction was then allowed to proceed at 42°C for 60 min, followed by additional heating at 95°C for 5 min to inactivate the enzyme. The resultant cDNA was either used immediately or stored at -20°C.

### Detection of HIV-1 RNA transcripts by RT-PCR

The cDNA synthesized from 1 μg total RNA isolated from trophoblastic cells was used for PCR amplification of HIV-1-*gag* sequences. To minimize misprimed DNA amplification following cDNA synthesis, the Hot Start PCR method was used[38]. After AmpliWax™ (Applied Biosystems) was added to tubes containing the cDNA, the tubes were heated at 80°C for 5 min, and then cooled down to 25°C for 5 min. The PCR master mixture (0.2 μM primers SK38 and SK39, 0.2 mM dNTP, 2 mM $MgCl_2$, PCR Buffer II, and 2.5 U AmpliTaq DNA polymerase) was prepared using RT-PCR core reagents supplied by Applied Biosystems, and aliquoted to each tube. The sequences for the HIV-1-*gag* specific primers SK38 and SK39 (Applied Biosystems) are 5′ATAATCC ACCTATCCCAGTAGGAGAAAT3′ and 5′ TT TGGTCCTTGTCTTATGTCCAGAATGC3′, respectively. After heating at 94°C for 5 min, the PCR was carried out for 35 cycles of 30 s at 94°C, 30 s at 55°C and 30 s at 72°C and immediately followed by a final extension at 72°C for 10 min using a thermal cycler (Model 9600; Perkin-Elmer Cetus, Norwalk, CT).

Two-fold serial dilutions, ranging from 5000 down to 156 copies, of HIV-1 positive control DNA and HIV-1 negative control DNA (Applied Biosystems) were also amplified by the PCR. The specificity of the HIV-1-*gag* sequence was verified by vacuum blotting the PCR product onto a nylon membrane (GeneScreen Plus; DuPont, Boston, MA), followed by hybridization with $^{32}P$-labeled SK19 oligonucleotide (Applied Biosystems). The Southern blot of the HIV-1-*gag* PCR products was visualized using a PhosphoImager (Molecular Dynamics, Sunnyvale, CA). Based on the signal intensity of the band, each sample was arbitrarily scored as having 'low' or 'high' copies of HIV-1-*gag* transcripts if the pixal value of the sample band was less than or greater than the number of HIV-1-*gag* transcripts in the highest dilution for the positive standard. Hence, samples with < 156 copies were scored as 'low' and samples with > 156 copies were scored as 'high'.

### Detection of IFN-β and β-actin messages by RT-PCR

One quarter of the cDNA derived from 1 μg of total RNA was used for the PCR of IFN-β and β-actin. The PCR mixture contained 0.125 μM upstream and downstream primers, 0.2 mM dNTP, 2 mM $MgCl_2$, PCR Buffer II, and 2.5 U

AmpliTaq DNA polymerase. The nucleotide sequences for each of the PCR primers were as follows: IFN-β sense primer, 5′GAACTTTG ACATCCCTGAGGAGATTAAGCAGC3′; IFN-β anti-sense primer, 5′GTTCCTTAGGA-TTTCCACTCTGACTATGGTCC3′; β-actin sense primer, 5′CTGTCTGGCGGCACCAC-CATG3′; and β-actin anti-sense, 5′CTCCTGC-TTGCTGATCCAC3′. The PCR products for IFN-β and β-actin were 352 and 189 bp, respectively. The IFN-β and β-actin was amplified for 35 cycles of denaturing at 94°C for 10 s, primer annealing at 60°C for 20 s and extension at 72°C for 20 s.

After PCR Southern blotting and hybridization were performed. The signal intensities of the PCR products of IFN-β and β-actin were obtained using a phosphoimager. Using the signal intensity of the β-actin band as a benchmark, the relative intensity (in arbitrary units) of the IFN-β was calculated and the results rendered as a ratio to the β-actin amplification.

### Statistical analysis

The nonparametric Mann–Whitney test was used to determine differences in the production of IFNs and β-chemokines and the level of IFN-β transcription by placental trophoblasts of HIV-1-infected and control women. The software package PRISM™ (GraphPad Software, Inc., San Diego, CA) was used to perform the statistical analyses.

### RESULTS

### Characteristics of study subjects

Twenty-five infants born to HIV-1-infected mothers had clinical follow-up visits for three years at the Pediatric AIDS Clinical Trials Unit in Houston and showed no evidence of HIV-1 infection (Table 1). The HIV-1-*gag* sequence was present in trophoblasts of all HIV-1-infected women. Based on the semi-quantitative RT-PCR, 12 of the 29 placental trophoblast preparations from HIV-1 infected women had < 156 copies of HIV-1-*gag* transcripts (Table 1).

### Constitutive levels of IFN-α, IFN-β, and IFN-γ produced by placental trophoblasts in culture

Constitutive levels of IFN-α, IFN-β, and IFN-γ were measured in culture supernatants of trophoblasts isolated from 29 HIV-1-infected and 9 control women. The median level of IFN-β constitutively produced by trophoblasts of HIV-1-infected women over a 24-h period was significantly higher than that of control women (Figure 1). The median constitutive levels of IFN-β for HIV-1-infected compared with control women were 3.5 pg/ml versus 3.1 pg/ml ($p$ = 0.0465) at 12 h and 3.7 pg/ml versus 3.1 pg/ml ($p$ = 0.0293) at 24 h, respectively. On the other hand, the median levels of IFN-γ and IFN-α constitutively produced by trophoblasts of HIV-1-infected and control women over the 24-h period were similar. The median constitutive levels of IFN-α produced by the trophoblasts of HIV-1-infected compared with control women were 4.2 pg/ml versus 4.4 pg/ml at 12 h and 4.6 pg/ml versus 4.8 pg/ml at 24 h, respectively. Similarly, the median constitutive levels of IFN-γ produced by the trophoblasts of HIV-1-infected and control women over a 24-h period were 17.8 pg/ml and 14.4 pg/ml at 12 h compared with 17.8 pg/ml and 11.5 pg/ml at 24 h, respectively.

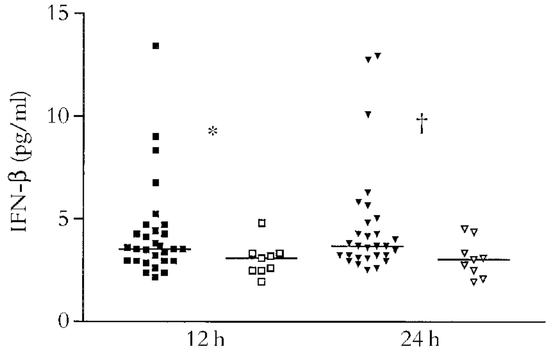

**Figure 1** Constitutive production of interferon-β by placental trophoblasts at 12-h and 24-h cultures (solid symbols: human immunodeficiency virus (HIV)-1-infected; open symbols: controls). There were significant differences in the median interferon (IFN)-β production between trophoblasts of HIV-1-infected and control women as determined by the Mann–Whitney test (two-tailed); *$p$ = 0.0465; **$p$ = 0.0293 for 12-h and 24-h cultures, respectively

There was no correlation between the amounts of interferons produced by trophoblasts and their HIV-1 burden.

### Transcription of IFN-β by placental trophoblasts

The level of IFN-β transcription was determined by RT-PCR in trophoblasts obtained from 13 HIV-1-infected and 10 control women. The median level of constitutive IFN-β transcription (as the ratio of signal intensity, IFN-β: β-actin) was significantly higher in HIV-1-infected trophoblasts compared with that of control women at 12 h (0.6 *vs* 0.1, $p = 0.0470$) and at 24 h (0.9 *vs* 0.5, $p = 0.0386$), respectively (Figure 2).

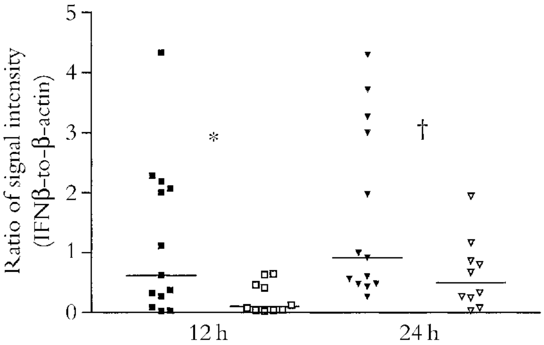

**Figure 2** Constitutive transcription of interferon (IFN)-β by placental trophoblasts (solid symbols: human immunodeficiency virus (HIV)-1-infected; open symbols: controls). There were significant differences in the level of IFN-β transcription between trophoblasts of HIV-1-infected and control women as determined by the Mann–Whitney test (one-tailed); $*p = 0.0470$; $^{\dagger}p = 0.0386$ for 12-h and 24-h cultures, respectively

### Production of β-chemokines by placental trophoblasts

Constitutive levels of MIP-1α, MIP-1β, and RANTES in culture supernatants of 17 HIV-1-infected and 7 control subjects were determined by enzyme-linked immunosorbent assay (ELISA). The medians of the constitutive levels of MIP-1α, MIP-1β, and RANTES produced by trophoblasts of HIV-1-infected women were 1025 pg/ml, 1236 pg/ml, and 160 pg/ml, respectively. The medians of the constitutive levels of MIP-1α, MIP-1β and RANTES produced by trophoblasts of control women were 1628 pg/ml, 355 pg/ml, and 359 pg/ml, respectively. Trophoblasts of HIV-1-infected women constitutively produced more MIP-1β and less MIP-1α and RANTES compared with those of the control women (Figure 3). However, there were no statistical differences in the constitutive production of these β-chemokines by trophoblasts of HIV-1-infected and control women. After 24 h in culture, trophoblasts of HIV-1-infected and control women produced comparable levels of β-chemokines (data not shown). There was no correlation between the amounts of β-chemokines produced by trophoblasts and their HIV-1 burden.

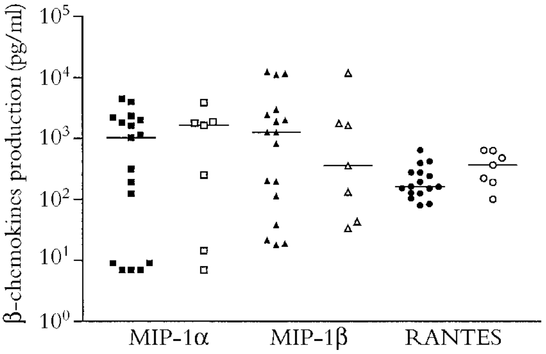

**Figure 3** Constitutive production of β-chemokines by placental trophoblasts at 12-h cultures (solid symbols: human immunodeficiency virus (HIV)-1-infected; open symbols: controls). MIP, macrophage inflammatory protein; RANTES, regulated on activation T cell expressed and secreted

### DISCUSSION

Placental trophoblastic cells are epithelial cells of fetal origin that closely resemble macrophages[20] and, as such, possess antiviral mechanisms similar to those of macrophages. For instance, infection of monocytes/macrophages with HIV-1 leads to the production of IFN-β, a type I IFN that is capable of inhibiting replication of HIV-1 in these cells[39]. However, infection of cells is not required for the induction of IFNs as treatment of cultured macrophages with a viral inducer such as HIV-1 rgp120 can lead to the predominant production of IFN-β by these cells[39] and protection against subsequent infection with the virus[40].

The level of IFN-α production by placental trophoblasts is dependent on the gestational age and the stage of differentiation of these cells, with first trimester trophoblasts producing 5–6 times more IFNs than third trimester trophoblasts[41,42]. Among the IFNs produced locally by the placenta during pregnancy[43], IFN-β is the predominant species and possesses more antiviral potential than IFN-α[40]. In the present study, trophoblasts isolated from term placentas of HIV-1-infected and control women produced equivalent levels of IFN-α. In contrast, trophoblasts from HIV-1-infected women produced significantly more IFN-β than trophoblasts of control women. The differential expression of IFN-β and not IFN-α by HIV-1-infected trophoblasts may be due, in part, to differences in transcriptional regulation, since the promoter of IFN-β, and not the promoter of IFN-α, contains an NF-κB binding site[44]. This difference, along with the induction of NF-κB by HIV-1[45], suggests that HIV-1 is a potent inducer of IFN-β.

Alternatively, IFN-β transcription can be induced by TNF-α[46], a cytokine previously shown to be present at elevated levels in trophoblasts of HIV-1-infected women[14]. Hence it is plausible that HIV-1 together with the HIV-1-induced TNF-α is capable of acting synergistically through the multipoietic transcription factor NF-κB to stimulate the production of IFN-β by HIV-1-infected trophoblasts. On the other hand TNF-α may directly or indirectly potentiate the antiviral activity of a type II IFN, IFN-γ, through the induction of IFN-β[46]. Indeed, the major component of the antiviral activity of IFN-γ may be due to its induction of IFN-α and IFN-β[47]. Within the placenta and throughout gestation, IFN-γ is produced by Hofbauer cells and syncytiotrophoblasts[48,49]. IFN-γ may protect the host cells from infection with HIV-1 by inhibiting binding of the virus with the coreceptor CCR5 on the target cell[50]. As part of its role in immune activation, IFN-γ induces 2′,5′-oligoadenylate (2-5A) synthetase activity to suppress HIV-1 replication in trophoblasts[51]. Nevertheless, we detected equivalent amounts of IFN-γ produced by trophoblasts of HIV-1-infected and control women and found that there was no correlation between IFN-γ production and the HIV burden of placental trophoblasts.

β-Chemokines can prevent cells from being infected with HIV-1 by blocking the interaction between the virus and the chemokine receptors on the cells[33,34]. Although RANTES and MIP-1α are capable of binding with more than one chemokine receptor, MIP-1β can only bind with CCR5[52]. A previous study detected no RANTES and only low levels of MIP-1α and MIP-1β in cultures of early trophoblasts either infected or uninfected with HIV-1[30]. Not only were RANTES, MIP-1α, and MIP-1β detected in cultures of term trophoblasts from HIV-1-infected women in the present study, but higher levels of MIP-1β, and not MIP-1α or RANTES, were produced by trophoblasts of HIV-1-infected women compared to control women (1236 *vs* 355 pg/ml) (Figure 3). The increase in MIP-1β production alone by term trophoblasts suggests that infection of the fetus with HIV-1 may be prevented by blocking of CCR5.

### CONCLUSIONS

Despite the up-regulation in the transcription and production of the inflammatory cytokines IL-1β, IL-6, and TNF-α by trophoblasts of HIV-1-infected women, in this study only a low level of HIV-1 replication was detected in term trophoblasts[14] which may be, in part, owing to the combined antiviral activity of the three IFN species[47]. It has been reported that only minute amounts of these proteins are required to prevent HIV-1 infection of target cells[39]. Alternatively, reduction in virus burden in 23 of the 29 HIV-1-infected pregnant women may be due to ZDV anti-retroviral therapy (Table 1). The exact mechanism by which trophoblast IFNs defend the fetus against infection with HIV-1 in the feto-placental unit is unclear. Some workers have suggested that IFNs can directly suppress HIV-1 replication[39,40] through transcriptional regulation[44] and down-regulation of CD4 receptor expression on susceptible cells[50]. However, the present study was unable to demonstrate an inverse correlation between the level of HIV-1-*gag* transcripts and the production of interferons by placental trophoblasts. Although the production of β-chemokines by trophoblasts of HIV-1-infected and control women were similar, these chemokines may act synergistically to control the spread of HIV-1 from maternal to fetal cells at the local level. Further studies are therefore warranted to establish the role of IFNs and β-chemokines in preventing transmission of HIV-1 from mother to fetus.

### ACKNOWLEDGEMENTS

This study was funded by grants AI41089, AI39131, and HD34031 from the National Institutes of Health, and General Clinical Research Center grant RR00188 from the National Center for Research Resources to Dr William T. Shearer.

The authors wish to thank Dr James Turpin of Southern Research Institute (Frederick, MD) for stimulating discussions, Stephen Li and De-Yu Shen for isolating the trophoblastic cells, and Hazel Dalton for the immunoperoxidase staining.

### REFERENCES

1. Fowler MG. Update: transmission of HIV-1 from mother to child. *Curr Opin Obstet Gynecol* 1997;9: 343–8
2. Amirhessami-Aghili N, Spector SA. Human immunodeficiency virus type 1 infection of human placenta: potential route for fetal infection. *J Virol* 1991;65:2231–6
3. Backe E, Jimenez E, Under M, *et al.* Demonstration of HIV-1 infected cells in human placenta by *in situ* hybridization and immunostaining. *J Clin Pathol* 1992;45:871–4
4. Sheikh AU, Polliotti BM, Miller RK. Human immunodeficiency virus infection: *in situ* polymerase chain reaction localization in human placentas after *in utero* and *in vitro* infection. *Am J Obstet Gynecol* 2000;182:207–13
5. Kesson AM, Fear WR, Kazazi F, *et al.* Human immunodeficiency virus type 1 infection of human placental macrophages *in vitro*. *J Infect Dis* 1993; 168:571–9
6. Kesson AM, Fear WR, Williams L, *et al.* HIV infection of placental macrophages: their potential role in vertical transmission. *J Leuk Biol* 1994;56: 241–6
7. Fear WR, Kesson AM, Naif H, *et al.* Differential tropism and chemokine receptor expression of human immunodeficiency virus type 1 in neonatal monocytes, monocyte-derived macrophages, and placental macrophages. *J Virol* 1998;72:1334–44
8. David FJE, Autran B, Tran HC, *et al.* Human trophoblast cells express CD4 and are permissive for productive infection with HIV-1. *Clin Exp Immunol* 1992;88:10–16
9. Kilani RT, Chang L-J, Garcia-Lloret MI, *et al.* Placental trophoblasts resist infection by multiple human immunodeficiency virus (HIV) type 1 variants even with cytomegalovirus coinfection but support HIV replication after provirus transfection. *J Virol* 1997;71:6359–72
10. Tscherning-Casper C, Papadogiannakis N, Anvret M, *et al.* The trophoblastic epithelial barrier is not infected in full-term placentae of human immunodeficiency virus-seropositive mothers undergoing antiretroviral therapy. *J Virol* 1999;73:9673–8
11. Reuben JM, Lee B-N, Popek EJ. HIV and the Placenta. In Shearer WT, Mofenson L, eds. *HIV and Women and Pregnancy*. Philadelphia: W.B. Saunders, 1998:371–400
12. Zachar V, Norskov-Lauritsen N, Juhl C, *et al.* Susceptibility of cultured human trophoblasts to infection with human immunodeficiency virus type 1. *J Gen Virol* 1991;72:1253–60
13. Zachar V, Ebbesen P, Thomas RA, *et al.* Basal and tat-transactivated expression from the human immunodeficiency virus type 1 long terminal repeat in human placental trophoblast rules out promotor-enhancer activation as the partial block to viral replication. *J Gen Virol* 1994;75:1461–8
14. Lee B-N, Ordonez N, Popek EJ, *et al.* Inflammatory cytokine expression is correlated with the level of human immunodeficiency virus (HIV) transcripts in HIV-infected placental trophoblastic cells. *J Virol* 1997;71:3628–35
15. Martin AW, Brady K, Smith SI, *et al.* Immunohistochemical localization of human immunodeficiency virus p24 antigen in placental tissue. *Hum Pathol* 1992;23:411–14
16. Mattern CF, Murray K, Jensen A, *et al.* Localization of human immunodeficiency virus core antigen in term human placentas. *Pediatrics* 1992;89:207–9
17. Boyer PJ, Dillon M, Navaie M, *et al.* Factors predictive of maternal-fetal transmission of HIV-1. Preliminary analysis of zidovudine given during pregnancy and/or delivery. *JAMA* 1994;271: 1925–30
18. Chandwani S, Greco MA, Mittal K, *et al.* Pathology and human immunodeficiency virus expression in placentas of seropositive women. *J Infect Dis* 1991;163:1134–8

19. Nelson AM, Firpo A, Kamengo M, *et al.* Pediatric AIDS and perinatal HIV infection in Zaire: epidemiologic and pathologic findings. *Prog AIDS Pathol* 1992;3:1–33
20. Guilbert L, Robertson SA, Wegmann TG. The trophoblast as an integral component of a macrophage-cytokine network. *Immunol Cell Biol* 1993;71:49–57
21. Nabel G, Baltimore D. An inducible transcription factor activates expression of human immunodeficiency virus in T cells. *Nature* 1987;326:711–13
22. Folks TM, Justement J, Kinter A, *et al.* Cytokine-induced expression of HIV-1 in a chronically infected promonocyte cell line. *Science* 1987;238: 800–2
23. Meylan PRA, Gratelli JC, Munis JR, *et al.* Mechanisms for the inhibition of HIV replication by interferon-α, -β and -γ in primary human macrophages. *Virol* 1993;193:138–48
24. Coccia EM, Krust B, Hovanessian AG. Specific inhibition of viral protein synthesis in HIV-infected cells in response to interferon treatment. *J Biol Chem* 1994;269:23087–94
25. Zdrakovic M, Knudsen HJ, Liu X, *et al.* High interferon alpha levels in placenta, maternal, and cord blood suggest a protective effect against intrauterine herpes simplex virus infection. *J Med Virol* 1997;51:210–13
26. Zachar V, Fazio-Tirrozzo G, Fink T, *et al.* Lack of protection against vertical transmission of HIV-1 by interferons produced during pregnancy in a cohort from East African republic of Malawi. *J Med Virol* 2000;61:195–200
27. Ahmad N. Maternal-fetal transmission of human immunodeficiency virus. *J Biomed Sci* 1996;3: 238–50
28. Zachar V, Zacharova V, Fink T, *et al.* Genetic analysis reveals ongoing HIV type 1 evolution in infected human placental trophoblast. *AIDS Res Hum Retrovir* 1999;15:1673–83
29. Zhu T, Mo H, Wang N, *et al.* Genotypic and phenotypic characterization of HIV-1 patients with primary infection. *Science* 1993;261:1179–81
30. Mognetti B, Moussa M, Croitoru J, *et al.* HIV-1 co-receptor expression on trophoblastic cells from early placentas and permissivity to infection by several HIV-1 primary isolates. *Clin Exp Immunol* 2000;119:486–92
31. Courgnaud V, Laure F, Brossard A, *et al.* Frequent and early *in utero* HIV-1 infection. *AIDS Res Hum Retrovir* 1991;7:337–41
32. Lairmore MD, Culhbert PS, Utley LL, *et al.* Cellular localization of CD4 in the human placenta. Implications for maternal-to-fetal transmission of HIV. *J Immunol* 1993;151:1673–81
33. Cocchi F, DeVico AL, Garzino-Demo A, *et al.* Identification of RANTES, MIP-1 alpha, and MIP-1 beta as the major HIV-suppressive factors produced by CD8+ T cells. *Science* 1995;270: 1811–15
34. Cocchi F, DeVico AL, Garzino-Demo A, *et al.* The V3 domain of the HIV-1 gp120 envelope glycoprotein is critical for chemokine-mediated blockade of infection. *Nat Med* 1996;2:1244–7
35. Taniguchi T, Matsuzaki N, Kameda T, *et al.* The enhanced production of placental interleukin-1 during labor and interuterine infection. *Am J Obstet Gynecol* 1991;165:131–7
36. Kauma SW, Walsh SW, Nestler JE, Turner TT. Interleukin-1 is induced in the human placenta by endotoxin and isolation procedures for trophoblasts. *J Clin Endocrinol Metab* 1992;75:951–5
37. McGann KA, Collman R, Kolson DL, *et al.* Human immunodeficiency virus type 1 causes productive infection of macrophages in primary placental cell cultures. *J Infect Dis* 1994;169:746–56
38. Chou Q, Russell M, Birch DE, *et al.* Prevention of pre-PCR mis-priming and primer dimerization improves low-copy-number amplifications. *Nucleic Acids Res* 1992;20:1717–23
39. Gessani S, Puddu P, Varano B. Role of interferons in the restriction of HIV replication in human monocytes/macrophages. *Res Immunol* 1994;145: 659–62
40. Gessani S,Puddu P,Varono B, *et al.* Induction of beta interferon by human immunodeficiency virus type I and its gp120 protein in human monocytes-macrophages. Role of beta interferon in the restriction of virus replication. *J Virol* 1994;68:1983–6
41. Aboagye-Mathiesen G, Toth FD, Petersen PM, *et al.* Differential interferon production in human first and third trimester trophoblast cultures stimulated with viruses. *Placenta* 1993;14:225–34
42. Paulesu L, Romagnoli R, Cintorino M, *et al.* First trimester human trophoblast expresses both interferon-gamma and interferon-gamma receptor. *J Reprod Immunol* 1994;27:37–48
43. Aboagye-Mathiesen G, Toth FD, Zdravkovic M, Ebbesen P. Human trophoblast interferons: production and possible roles in early pregnancy. *Early Pregnancy* 1995;1:41–53
44. MacDonald NJ, Kuhl D, Maguire D, *et al.* Different pathways mediate virus inducibility of the human IFN-α-1 and IFN-β genes. *Cell* 1990; 60:767–79
45. Hiscott J, Alper D, Cohen L, *et al.* Induction of human interferon gene expression is associated with a nuclear factor that interacts with the NF-kappa B site of the human immunodeficiency virus enhancer. *J Virol* 1989;63:2557–66
46. Hughes TK, Kasper TA, Coppenhaver DH. Synergy of antiviral actions of TNF and IFN-gamma: evidence for a major role of TNF-induced IFN-beta. *Antiviral Res* 1988;10:1–9
47. Hughes TK, Baron S. Do the interferons act singly or in combination? *J Interferon Res* 1987;7:603–14
48. Berkowitz RS, Faris HMP, Hill JA, Anderson DJ. Localization of leukocytes and cytokines in chorionic villi of normal placentas and complete hydatidiform moles. *Gynecol Oncol* 1990;37: 396–400
49. Bulmer JN, Morrison L, Johnson PM, Meyer A. Immunohistochemical localization of interferons in human placental tissues in normal, ectopic and molar pregnancy. *Am J Reprod Immunol* 1990;22: 109–16
50. Hariharan D, Douglas SD, Lee B, *et al*. Interferon-gamma upregulates CCR5 expression in cord and adult blood mononuclear phagocytes. *Blood* 1999; 93:1137–44
51. Zhang GY, Beltchev B, Fournier A, *et al*. High levels of 2',5'-oligoadenylate synthetase and 2',5'-oligoadenylate-dependent endonuclease in human trophoblast. *AIDS Res Hum Retrovir* 1993;9: 189–96
52. Garzino-Demo A, Moss RB, Margolick JB, *et al*. Spontaneous and antigen-induced production of HIV-inhibitory-chemokines are associated with AIDS-free status. *Proc Natl Acad Sci USA* 1999; 96:11986–91

RECEIVED 12/15/00; ACCEPTED 03/29/01
